# Spatiotemporal trends in WBGT and affected population in the MENA region (1951–2021)

**DOI:** 10.1038/s41598-026-54062-6

**Published:** 2026-06-14

**Authors:** Mohammed Magdy Hamed, Mohammed Rady, Zafar Iqbal, Mohamed Salem Nashwan, Shamsuddin Shahid

**Affiliations:** 1 Construction and Building Engineering Department, College of Engineering and Technology, Arab Academy for Science, Technology and Maritime Transport (AASTMT), B 2401 Smart Village, Giza, 12577 Egypt; 2 School of Civil & Environmental Engineering (SCEE), NUST, National University of Sciences and Technology, Sector H-12, Islamabad, 44000 Pakistan; 3 Department of Construction and Building Engineering, College of Engineering and Technology, Arab Academy of Science, Technology, and Maritime Transport (AASTMT), Elhorria, Cairo, 2033 Egypt; 4 Regional Climate Change Center, National Center for Meteorology, Al Warood District, Jeddah, Saudi Arabia

**Keywords:** Trends, Affected area, Human thermal exposure, Modified Mann–Kendall, ERA5, WBGT, Climate sciences, Environmental sciences

## Abstract

Rising temperatures and changes in other meteorological factors have significantly impacted human thermal comfort worldwide. Among global climate change hotspots, the Middle East and North Africa (MENA) have experienced a particularly rapid temperature rise compared to many other regions. Despite its vulnerability, long-term assessments of spatiotemporal heat stress trends and the affected population in MENA remain limited. This study evaluates changes in thermal stress using the wet-bulb globe temperature (WBGT) index derived from ERA5 meteorological data (0.25° resolution) from 1951 to 2020. The findings revealed that the average WBGT in the MENA region increased by 0.75–2.20 °C when comparing 2011–2020 with the 1951–1960 baseline. The most substantial increase occurred in the Arabian Peninsula, specifically Saudi Arabia, where the rise exceeded 0.4 °C per decade in most regions. Consequently, the annual frequency of “Normal conditions” days decreased by approximately 50 days, while high-risk and extreme heat-stress days increased by a corresponding 40 days across most of the region, particularly in the east. Significant trend analysis reveals a 10-day increase per decade across eastern and western MENA under extreme conditions. This rise has led to an additional 1.23 million people experiencing extreme heat stress for at least one day each year. The findings of this study can be useful to policymakers and researchers concerned with extreme heat in the MENA region.

## Introduction

The most certain and near-future consequence of global warming is an increase in the frequency of severe heat events^1,2^. Excessive exchange between the environment and the human body causes heat stress and affects public health, including heat-related illnesses such as heat exhaustion and heatstroke^3–5^. Moreover, heat can indirectly affect mental health and well-being, as exposure to extremely high temperatures may cause tension and anxiety and disrupt sleep^6,7^. Heat increase is injurious to susceptible species and disrupts reproductive cycles, thereby affecting the ecosystem and biodiversity and resulting in habitat loss^8^. In this respect, more frequent heat events, particularly in arid areas, can be quite harmful to plant growth, alter the time course of flowering and fruiting, and increase fire risk^9,10^. Through reduced crop yields, deterioration of animal health, and rising irrigation water requirements, heat events can negatively affect agricultural production and food security^4,11^. Severe economic impacts also result from heat surges. It can result in lowered labour productivity^12,13^, raised ventilation energy use, higher health costs, and structural damage^14,15^. Agriculture, tourism, and outdoor occupations may be severely affected^16,17^. Therefore, assessing the changes in heat events is vital for people’s well-being and the national economy.

Temperature is the principal constituent of heat^18^. However, it is influenced by several meteorological and environmental factors in addition to temperature. Higher humidity, for instance, can make the air feel hotter because it reduces the body’s ability to cool by evaporation of sweat. Wind speed can influence the perceived temperature by enhancing or reducing heat transfer from the body. Therefore, assessing heat-related impacts needs a comprehensive analysis of these factors. Several studies attempted to develop an index to describe human exposure to heat. Nevertheless, assessing thermal exposure and how this translates into psychological and physical stress is not easy, as there are many variables to consider, including individual characteristics such as age, gender, and health condition, as well as financial and psychological factors. The Wet-bulb Globe Temperature (WBGT) integrates meteorological and psychrometric parameters based on the heat exchange between the human body and the environment^19^. It has been reported that WBGT improves the evaluation of public health, labour activities, human activities, and environmental sustainability^20,21^. Furthermore, it can suggest heat-related danger levels and limited human physical activity^22,23^.

Many studies have used WBGT to assess regional trends in heat stress. Houmsi et al.^25^ investigated the changes that occur in WBGT in peninsular Malaysia and found that the trends for nighttime and daytime WBGT were positive at 0.24 and 0.11 °C per decade, respectively. Li et al.^26^ examined how WBGT has varied across China and found that, in dry western China, extremely high summer WBGT occurred nearly 1,000 times more often during the 2010s than during 1961–1990. Wang and Sun^27^ analyzed the highest daily maximum WBGT across China from 1961 to 2017 and found a significant increase. According to an analysis of WBGT trends conducted by Kyaw et al.^20^, the average WBGT during South Asia’s pre-monsoon hot season has been rising by 0.1–0.7 °C per decade, leading to a 66% increase in the number of days with dangerously high temperatures between 1979 and 2020. According to calculations by Li et al.^28^, the probability of a summer WBGT in the Northern Hemisphere in 2016 is 70 times higher than it was from 1973 to 2012. Excess of severe WBGT thresholds was evaluated by Willett and Sherwood^29^ across 15 locations and was shown to be rising globally, except in certain areas of the United States and Australia. According to research, areas with more frequent temperature extremes also have higher WBGT and more heat-stress days.

The Middle East and North Africa (MENA) region experienced the highest rise in extreme temperatures. The following studies assess temperature extremes, humidity, and wind speeds. However, a harmonised trend analysis of WBGT across the MENA region using high-resolution reanalysis data is still lacking. In Iraq, which is part of the MENA region, the trend in daily maximum temperatures has increased by 0.25–1.01 °C/decade, according to Salman et al.^30^, which is sevenfold higher than the world average. An analysis by Ntoumos et al.^31^ revealed an increase in the hottest-day temperature over the MENA region of 0.3–0.4 °C per decade. According to Zittis et al.^32^ super and ultra-extreme heatwave situations will occur in MENA in the near future. Recent regional climate modelling for the CORDEX-MENA domain highlights this path, projecting that warm spell durations could increase by more than 200 days per year under high-emission scenarios by the end of the century^33,34^. Numerous other studies have also alerted to an abrupt rise in the frequency and severity of weather extremes. As Lelieveld et al.^35^ state, under "business as usual", the annual maximum temperature of 43 °C in present times may easily rise above 50 °C toward the end of the century. Some regions of MENA will experience over 180 hot days per year between 2071 and 2100, with maximum extreme temperatures projected to reach at least 56°C, according to research by Ntoumos et al.^36^. Even if global warming is kept to 2 °C, the risk of death due to severe temperatures is expected to be three to seven times higher by the end of this century, as stated by Ahmadalipour et al.^37^. Even if the global mean temperature rose by 2 °C, Dosio and Fischer^38^ found that the number of people negatively impacted by temperature extremes will rise dramatically. The sharp rise in temperature and related extremes has certainly increased the severity of heat events and human thermal stress. However, no study has assessed the changes in such events. Only one study assessed the possible increase in WBGT in the region for future periods using regional climate models^39^. They reported a future increase in extreme WBGT above the critical threshold for human tolerance. Understanding ongoing changes is a primary prerequisite for assessing the impact of climate change on heat stress and formulating adaptation measures.

The above finding, along with the globally recognised MENA region as a global climate hotspot due to structural water scarcity, the convergence of climatic and meteorological stress, and the arid-to-semiarid climatic conditions, as reported by IPCC^40^, and the sensitivity of water-energy-food to climate change and a growing population^41,42^, makes the MENA region ideal for this study. While global studies have conducted heat stress analyses to evaluate WBGT at global and selected regional scales^3,44–46^, a harmonised, high-resolution, region-wide assessment of observed WBGT trends and population exposure across the entire MENA domain is lacking.

Therefore, this work focuses on assessing spatiotemporal variations in outdoor WBGT across regions and populations at risk exposed to various heat levels in the MENA region. It utilised the Liljegren method^43^ to estimate WBGT using the latest ERA5 meteorological datasets and the affected population using the LandScan population dataset. Specifically, this study provides: (i) a comprehensive spatiotemporal synthesis of extreme heat across the MENA region, introducing the Diurnal WBGT Range to quantify the critical loss of nighttime thermal relief using ERA5 reanalysis data; (ii) a unified, high-resolution regional baseline that leverages a physically robust WBGT formulation (the Liljegren method) to capture the complex radiation and wind dynamics often missed by simpler indices in arid climates; (iii) quantitative tracking of the specific geographical areas transitioning into higher heat-stress zones over time; and (iv) a normalized assessment of population exposure that successfully isolates the true climate-driven risk from the region’s underlying demographic growth using high-resolution LandScan data. Such results can help raise awareness among vulnerable populations and inform regional adaptation planning. The spatial distribution of WBGT and the trend maps resulting from this study are important resources for policymakers and researchers seeking to mitigate the impacts of extreme heat and develop effective strategies to reduce adverse effects in the MENA region.

## Area description and data

### MENA region geography

**Fig. 1:**
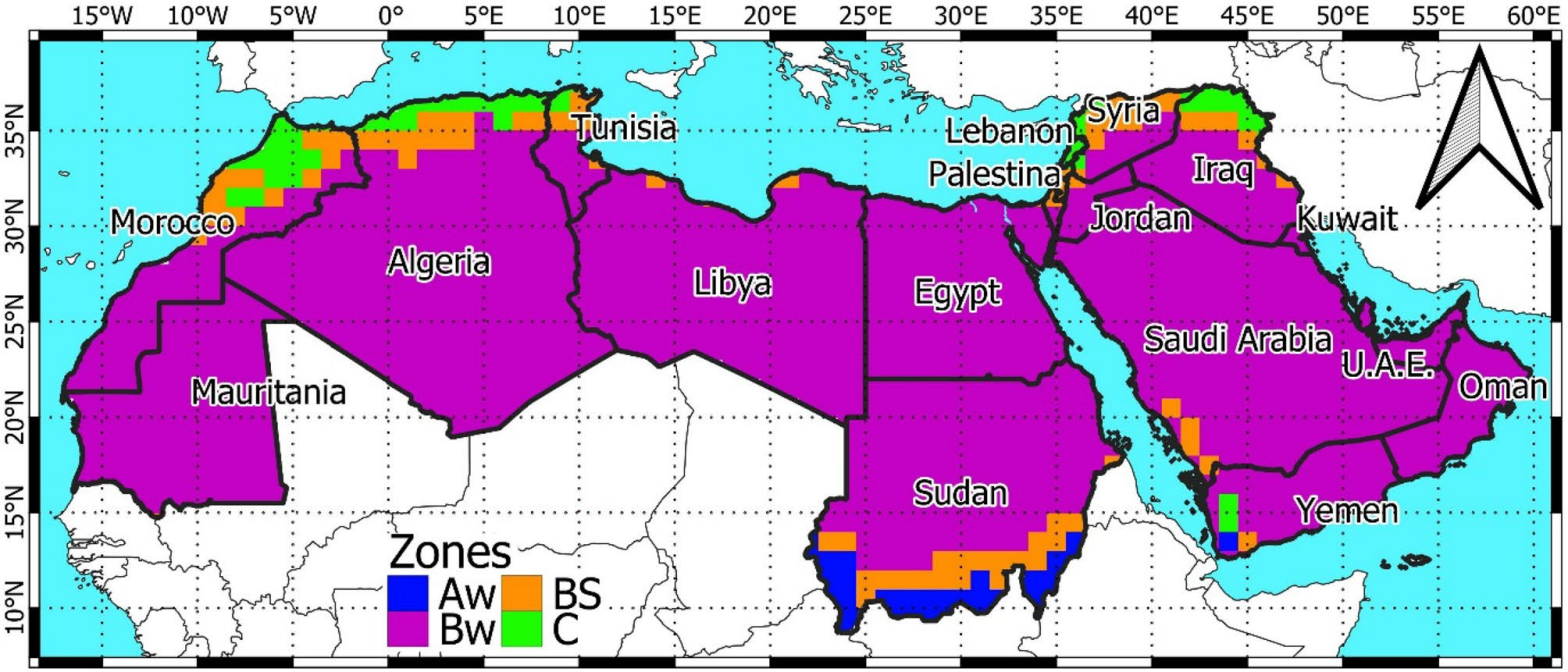
Study area location with its climate zones based on the Köppen climate classification.

The MENA region includes 20 nations bounded by 9°N-38°N and 17°W-60°E (Fig. 1). It covers an area of 13.3 million km^2^ and has nearly half a billion people. It contains five maritime ecosystems (i.e., oceanic realms), five zoological regions, and four coastal regions. The annual precipitation in MENA ranges from 0 to 1000 mm, and the average temperature range is from − 5 to 47 °C. Due to the extreme dryness of the terrain, almost two-thirds of MENA is plagued by water scarcity and desertification^44^. The MENA nations share similar characteristics, including arid climates, short rainy seasons, and year-round high temperatures. Thus, noticeable climate change is a pervasive phenomenon across the MENA region. Based on the Köppen classification system, the region is composed of six climatic zones: mild temperature dry winter (Cw), mild temperature dry summer (Cs), mild temperate fully humid (Cf), dry arid desert (BW), dry arid steppe (BS), and tropical Savannah climate (Aw)^45^. Due to their relatively small total area, all mild arid regions are incorporated into zone C. The BW zone covers 85% of the region’s geographic area and has annual precipitation ranging from 0 to 100 mm. During the winter, temperatures can drop to as low as 6 °C, particularly in climate zone C. Summer temperatures in the BS and BW zones typically climb above 40 °C. Monthly precipitation in the Aw zone varies between 0 and 200 mm, while the BS and BW zones get a maximum monthly precipitation of 50 mm^45^.

### Data section

In this study, ERA5 is used to estimate WBGT, a global atmospheric reanalysis dataset compiled by the European Centre for Medium-Range Weather Forecasts (ECMWF)^46^. It is rated among the most accurate global reanalysis datasets due to advanced data assimilation techniques and a high-resolution numerical weather prediction model. It represents an integrated view of the Earth’s atmosphere, land surface, and oceans from 1940 to the present, with hourly data on a 0.25-degree grid. This is equivalent to approximately 31 km at the equator and is generated using a state-of-the-art numerical weather prediction model run in reconsidering mode, forced with a variety of observations from sources such as satellites, weather stations, and buoys. The resulting dataset is an excellent archive of past weather conditions, and ERA5 finds wide application in climate research and weather forecasting. A wide range of variables, such as temperature, wind speed, precipitation, and atmospheric pressure, is available in ERA5. Table 1 presents the important variables used in this study. In this study, the available hourly dataset was aggregated to a daily time scale, which was appropriate for the study’s aim.

**Table 1:** Main variables considered in the present study.

Symbol	Variable	Unit
u	Wind speed’s u-component at 10 m	ms^−1^
v	Wind speed’s v-component at 10 m	ms^−1^
T_d_	Dewpoint temperature at 2 m level	°C
T_a_	Air temperature at 2 m level	°C
P	Surface pressure	Pa
r_ss_	Surface net solar radiation	Jm^−2^
r_ssd_	Downward surface net solar radiation	Jm^−2^
r_st_	Surface net thermal radiation	Jm^−2^
r_ds_	Total sky direct solar radiation at the surface	Jm^−2^

ERA5 was selected for its high resolution and its reliability across diverse climatic regions^47–49^. Previous studies have highlighted ERA5 as a superior reanalysis product. For instance, Khadka et al.^47^ demonstrated its high performance in the Asian region, while Zuluaga et al.^49^ confirmed its accuracy in simulating radiation and temperature variables. Furthermore, Beck et al.^48^ noted that ERA5 consistently outperforms other global reanalysis datasets in capturing observed climate patterns. These favourable attributes make ERA5 the most robust available tool for assessing long-term heat stress trends in the MENA region. Also, hourly ERA5 data were used in several studies to calculate WBGT^20,25,50–52^.

The following research uses the population grid of the LandScan Global population dataset developed by Oak Ridge National Laboratory (ORNL)^53^ to calculate the population exposed to different heat levels. The annual population data have a spatial resolution of 30 arc-seconds (~ 1 km). LandScan is updated annually to reflect changes in the global population and political boundaries. It is a valuable resource for researchers, educators, humanitarians, and corporations worldwide. LandScan data was aggregated using the R packages "r.raster" and "r.terra" to achieve spatial consistency with ERA5. Population counts were computed as the weighted sum of all contributing grid cells that were not marked as NA. The dataset can be found here: https://landscan.ornl.gov/.

## Method

**Fig. 2:**
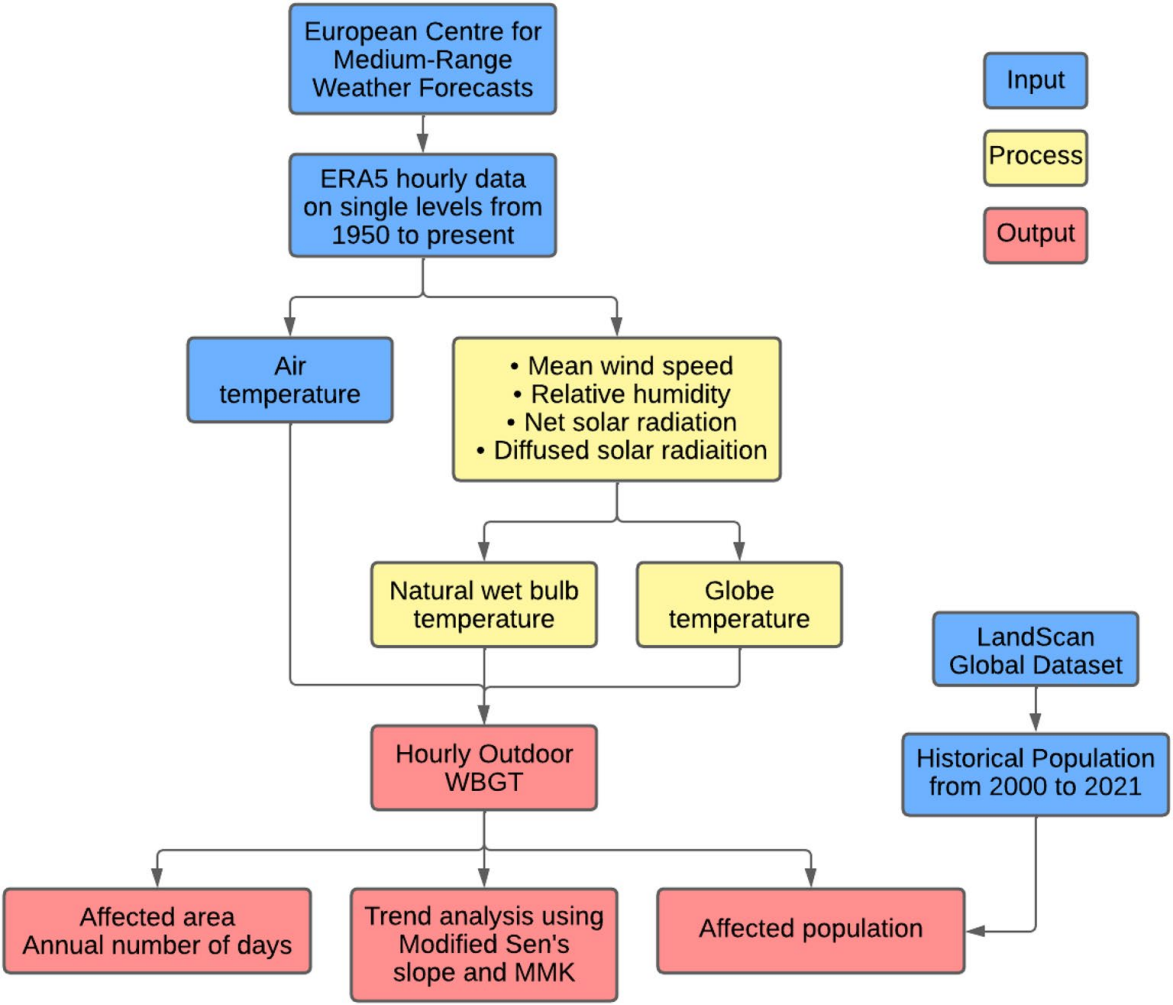
Flowchart showing the research procedure followed to assess spatiotemporal trends in WBGT and the affected population in the MENA region.

The present study followed the steps shown in the flowchart of Fig. 2 to achieve its aim. Initially, ERA5 data were used to determine the natural wet-bulb temperature and globe temperature. Subsequently, the minimum, mean, and maximum daily WBGT were computed from hourly WBGT and categorised into various heat levels (as detailed in Table A1 in the appendix) at each grid point using the Liljegren method^43^ (formulated in Eq. 1–3).

1 $$WBGT=0.7\times Tw+0.2\times Tg+0.1\times Ta$$

2 $${T}_{w}={T}_{a}-\frac{\Delta H}{{c}_{p}}\frac{{M}_{H2O}}{{M}_{Air}}\left(\frac{{P}_{r}}{{S}_{c}}\right)\left(\frac{{e}_{w}-{e}_{a}}{P-{e}_{w}}\right)+\frac{\Delta {F}_{net}}{Ah}$$

3 $${T}_{g}^{4}=\frac{1}{2}\left(1+{\varepsilon}_{a}\right){T}_{a}^{4}-\frac{h}{{\varepsilon}_{g}\sigma }\left({T}_{g}-{T}_{a}\right)+\frac{S}{2{\varepsilon}_{g}\sigma }\left(1-{\alpha}_{g}\right)\left[1+\left(\frac{1}{2cos\left(\theta \right)}-1\right){f}_{dir}+{\alpha}_{sfc}\right]$$

Here, Tw refers to the estimated temperature of the wet bulb in natural conditions of wind and sun, Tg is the temperature derived from the sun’s short-wave radiation and the soil’s long-wave radiation, where ${T}_{a}$ is dry bulb (ambient) temperature, $\Delta H$ is the heat of vaporisation, ${M}_{H2O}$ is the molecular weight of water vapour, ${c}_{p}$ is the specific heat at constant pressure, ${M}_{Air}$ is the molecular weight of dry air, ${S}_{c}$ is Schmidt number, ${P}_{r}$ is the Prandtl number, ${e}_{w}$ is emissivity wick, ${e}_{a}$ is emissivity air, $P$ is barometric pressure, $A$ is the wick’s surface, $\Delta {F}_{net}$ is the net radiation gain induced by the environment on the wick, and $h$ is the convective heat transfer coefficient, where ε_g_ = 0.95, α_g_ = 0.05, and α_sfc_ = 0.45, ε_a_ = atmospheric emissivity^54^ (function of T_d_ and T_a_), σ = Stefan-Bolzmann constant, h = the convective heat transfer coefficient, S = the solar constant, θ = the solar zenith angle, and f_dir_ = proportion of direct solar radiation.

Since ERA5 wind speed components (${u}_{10m}$ and ${v}_{10m}$) are provided at a 10 m height, a logarithmic wind profile was applied to estimate the hourly wind speed at the 2 m level required for WBGT calculations^55^. The wind speeds at 10 m and 2 m are presented as, where z is 10 m.

4 $${WS}_{10m}=\sqrt{{{u}_{10m}}^{2}+{{v}_{10m}}^{2}}$$

5 $${WS}_{2m}={WS}_{10m}\times \left(\frac{4.87}{\mathrm{l}\mathrm{n}(67.8\times z-5.42)}\right)$$

Sen’s slope method was applied to estimate the trends in maximum, minimum, and mean WBGT at each grid point. The significance of those trends was examined through the modified Mann–Kendall test developed by^56^.

Finally, the total population affected by each heat category was calculated. In this study, population exposure was defined as the total number of people residing in grid cells who experienced at least 1 day per year in a given WBGT heat category. For each grid cell, annual WBGT category counts were first determined. The gridded population was overlaid on the WBGT grid, and exposure was calculated as the number of individuals potentially exposed to a given heat-stress level per year.

## Results

**Fig. 3:**
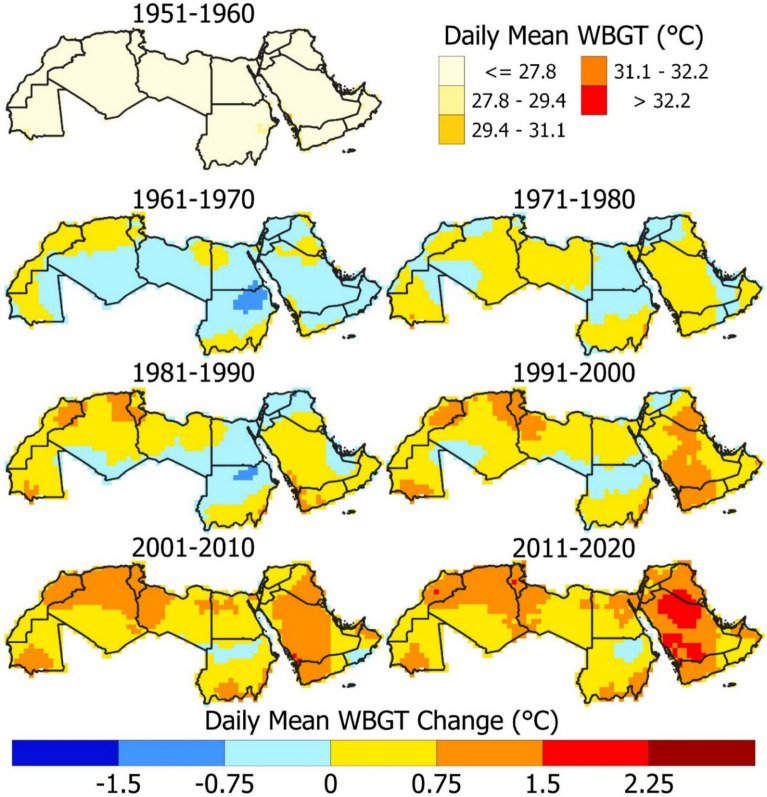
Spatial distribution of the historical daily mean WBGT (base period 1951–1960) and the absolute change in the following decades (1961 to 2020) in the MENA region.

**Fig. 4:**
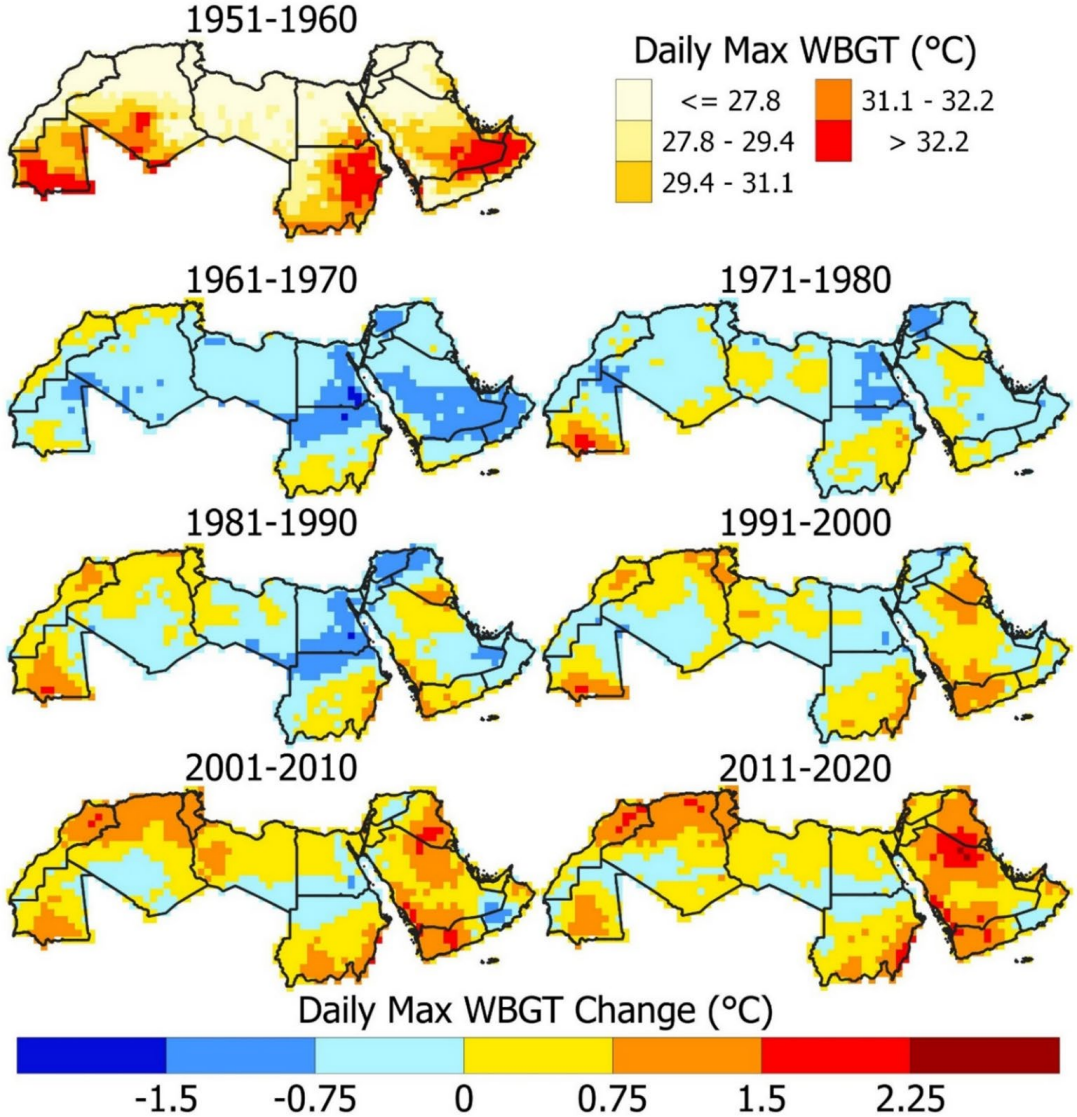
Same as Fig. 3, but for daily maximum WBGT.

Figures 3 and 4 depict the spatial changes in daily mean and maximum Wet Bulb Globe Temperature (WBGT) in the MENA region from 1951 to 2020, while the minimum WBGT is presented in the appendix. The first decade (1951–1960) serves as the reference period, against which changes in WBGT in subsequent decades are compared to demonstrate relative changes over time. The figures utilise five colours to represent different WBGT categories based on the heat conditions outlined in Table A1 in the appendix. Figure 3 illustrates the changes in mean WBGT over time. In the reference period, most climatic zones in MENA had low mean WBGT values below 27.8 °C. However, in the 1961–1970 period, it decreased by 0.75 °C in most zones. After the 2000s, the mean WBGT began to rise, particularly in North African and Arab Gulf nations in the eastern part, where the increase became 1.5 to more than 2.25 °C.

Figure 4 focuses on the changes in daily maximum WBGT. In the reference decade, the southern parts of MENA experienced extremely high daily maximum WBGTs. However, during the 1961–1990 period, most climatic zones had lower maximum WBGT values than in the reference decade. Subsequently, the daily maximum WBGT started to increase across most areas after 2001. However, the rate of increase in daily maximum WBGT for the region was much less than that of the minimum WBGT.

During the reference decade (Figure S1), all climatic zones in the MENA region maintained normal conditions, as indicated by the annual mean minimum WBGT being below 27.8 °C. In later decades, the minimum WBGT increased across most zones, particularly in the last two decades (2001–2020). The most significant increase was observed on the eastern side of MENA, encompassing Saudi Arabia and Jordan, with an increase of 2.25 °C between 2011 and 2020. Conversely, Sudan experienced relatively cooler conditions than the reference decade, with a 1.5 °C decrease in minimum WBGT.

**Fig. 5:**
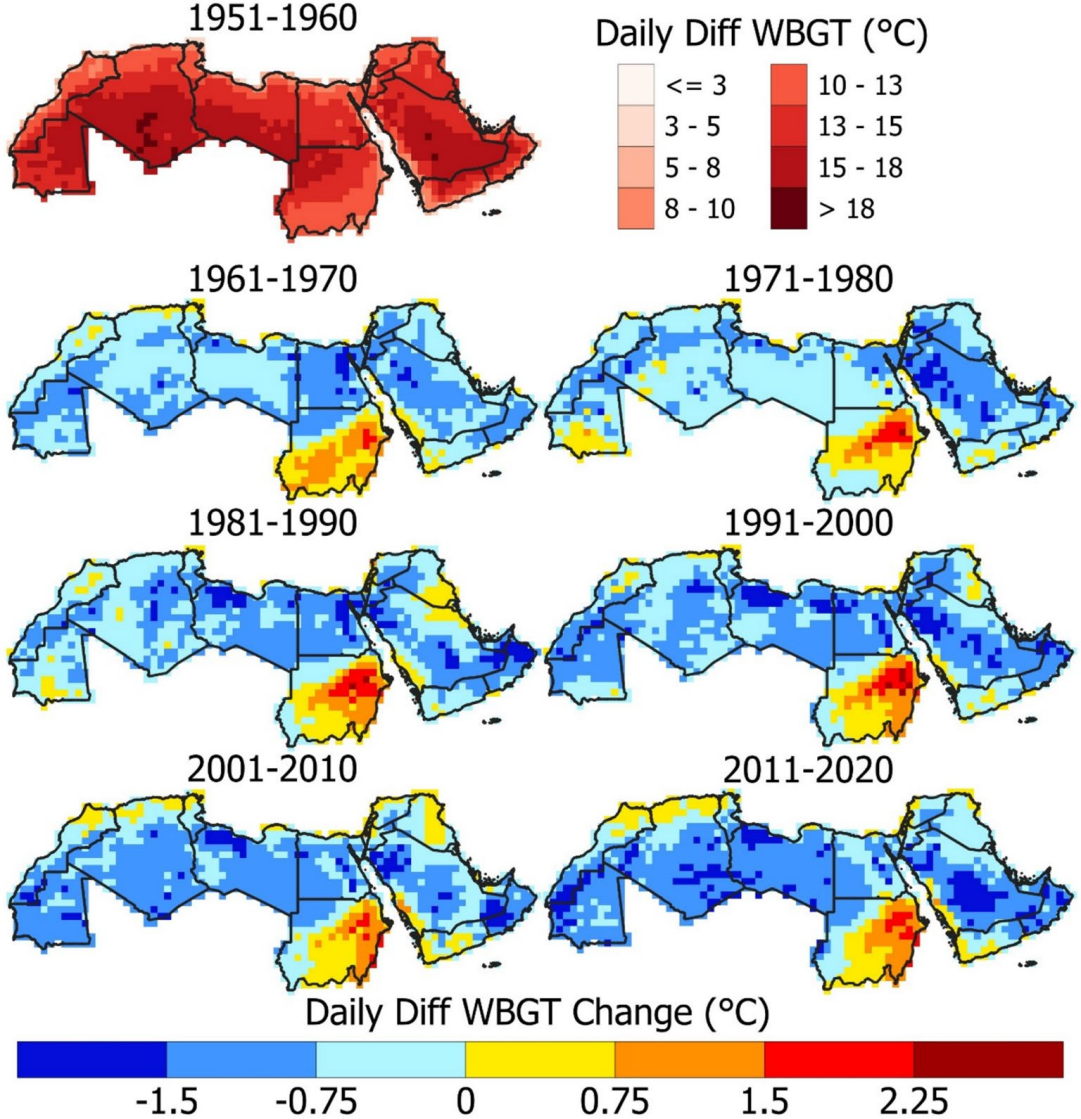
Spatial distribution of the historical daily diurnal WBGT range (calculated as the daily maximum minus the daily minimum WBGT) for the baseline period (1951–1960) and the absolute change in subsequent decades (1961–2020) across the MENA region.

Figure 5 combines the maximum and minimum WBGT by illustrating the spatiotemporal evolution of the diurnal WBGT range. Between 1951 and 1960, the baseline diurnal range was highly pronounced across the deep Saharan and the central Arabian Peninsula, exceeding 15 °C. However, data from subsequent decades show this gap shrinking sharply across the region. The diurnal WBGT range has progressively decreased, with notable reductions between − 0.75 and − 1.5 °C becoming widespread by the 2011–2020 period. This shrinking gap indicates that nighttime WBGT are rising faster than daytime WBGT. As a result, the region is losing the cooler nights that are biologically necessary for people to recover from daytime heat stress. An exception to this pattern appears in the south, primarily in Sudan, where the difference increased by up to 2.25 °C.

**Fig. 6:**
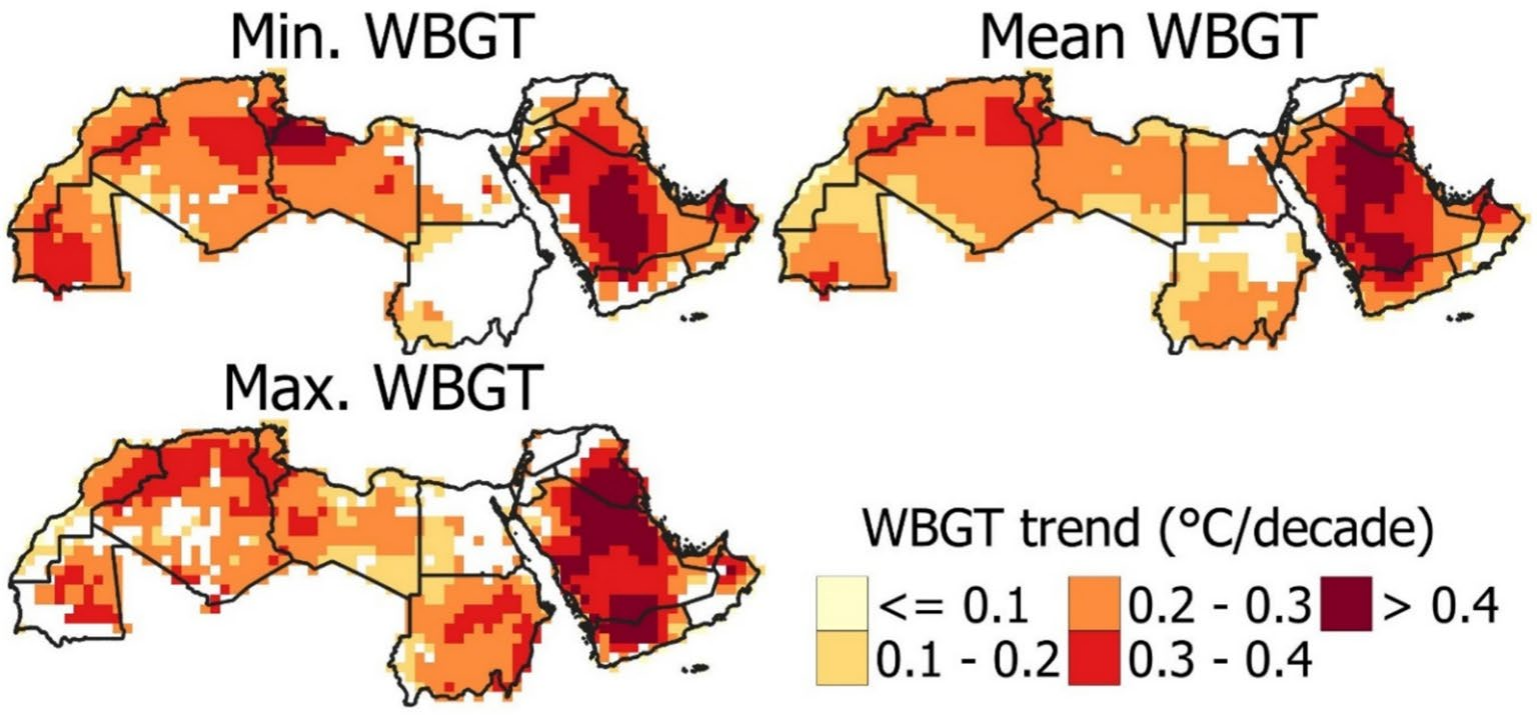
Minimum, mean, and maximum WBGT significant trends (*P* < 0.05) in the MENA region using Sen’s slope and MMK.

Figure 6 presents the significant trends in daily minimum, mean, and maximum WBGT in MENA from 1951 to 2020. The colours in the figure indicate the magnitude of change in WBGT per decade, with white representing insignificant changes. The results indicate that the variations in minimum WBGT over decades were relatively minor in Egypt, Sudan, and Syria. Conversely, the most significant changes in minimum, mean, and maximum WBGT were in the eastern part of MENA, surpassing 0.4 °C/decade.

**Fig. 7:**
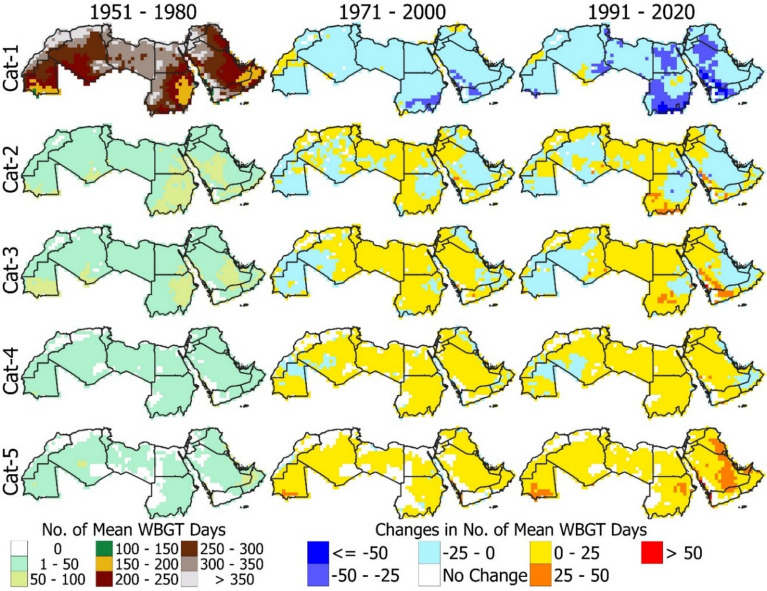
Annual number of mean WBGT days for each category in the MENA region.

**Fig. 8:**
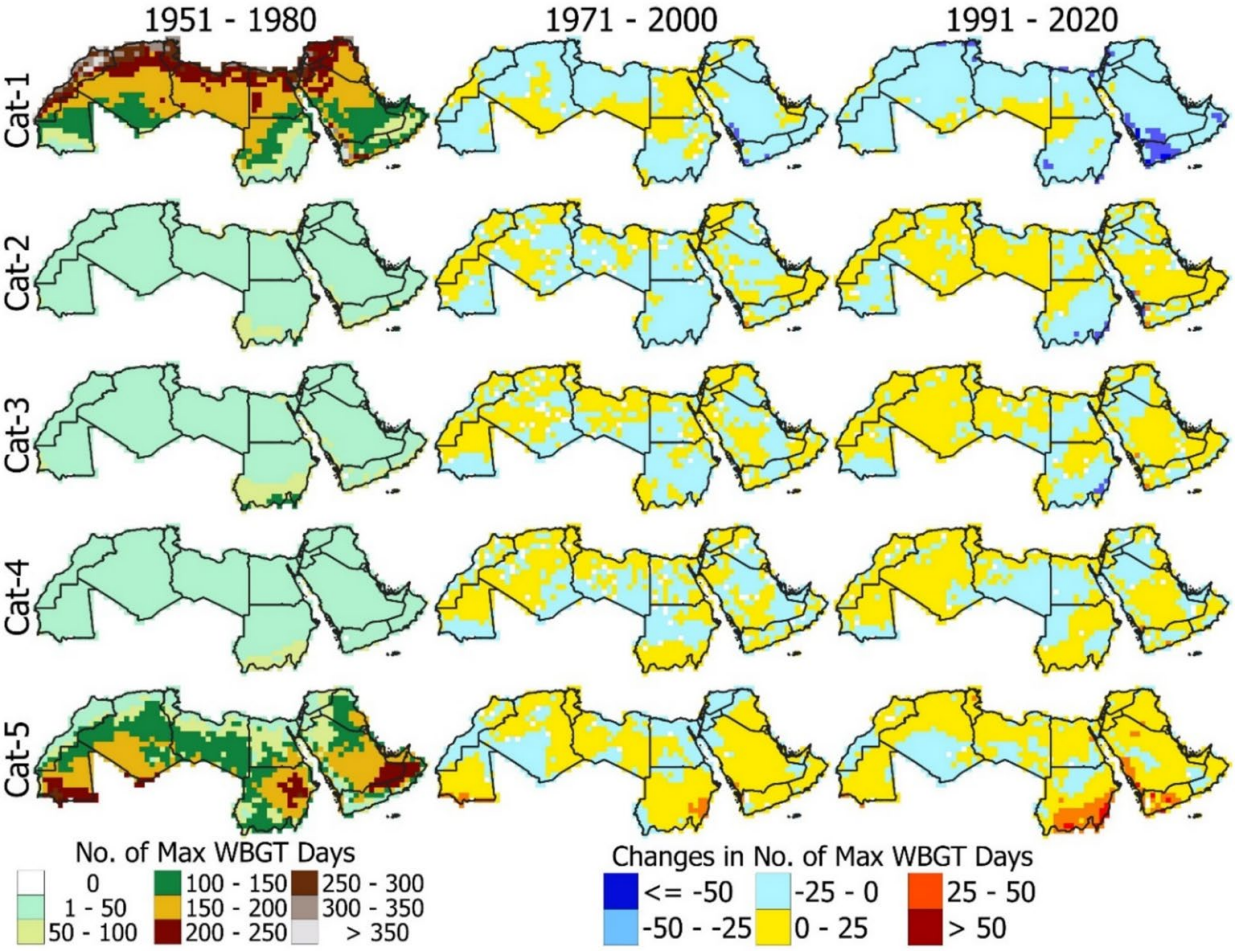
Annual number of maximum WBGT days for each category in the MENA region.

Figures 7 and 8 depict the annual number of days falling into different WBGT categories based on daily mean and maximum values, while minimum values are presented in Fig. A2 in the appendix. The historical study period (1951–2020) is divided into three thirty-year periods, with each column representing a period and each row representing a WBGT category. The first period (1951–1980) serves as the reference, against which changes in the number of days in each WBGT category for the other two periods are compared.

Figure 7 provides insights into the distribution of mean WBGT categories across MENA countries during different periods. Most MENA countries experience normal conditions (Category 1) over 200 days annually. Meanwhile, the number of days with mean WBGT in Categories 2–5 ranged from 0 to 100. In the subsequent periods, the number of days with mean WBGT in Category 1 decreased for all MENA nations, while the number of days in Categories 2–5 increased. The increase was 25–50 days for extreme heat-stress conditions in the eastern MENA.

Figure 8 focuses on the maximum WBGT categories in the reference period and other decades. Most MENA nations had normal conditions over 150 annual days, especially in the northern parts, where the number exceeded 200 days. The number of days with maximum WBGT in Categories 2–4 ranged from 0 to 50 during the same period, while Category 5 had over 100 days. In the following periods (1971–2020), the days with normal conditions gradually decreased while the severe and extreme heat stress conditions gradually increased.

Figure S2 shows that during the first period, most regions had a low minimum WBGT (< 27.8 °C). Only a few MENA nations were classified in categories 2 and 3, representing annual numbers of days ranging from 0 to 50 with moderate-risk conditions. Almost no nations fell into categories 4 and 5 during this period. In the second and third periods, WBGT exhibited similar patterns of change in minimum WBGT. For categories 1–3, the number of days relative to the first period ranged from − 25 to 25 for certain nations, including eastern Sudan and Mauritania. However, there was little to no change in the number of days falling into categories 4 and 5 for the minimum WBGT compared to the first period.

**Fig. 9:**
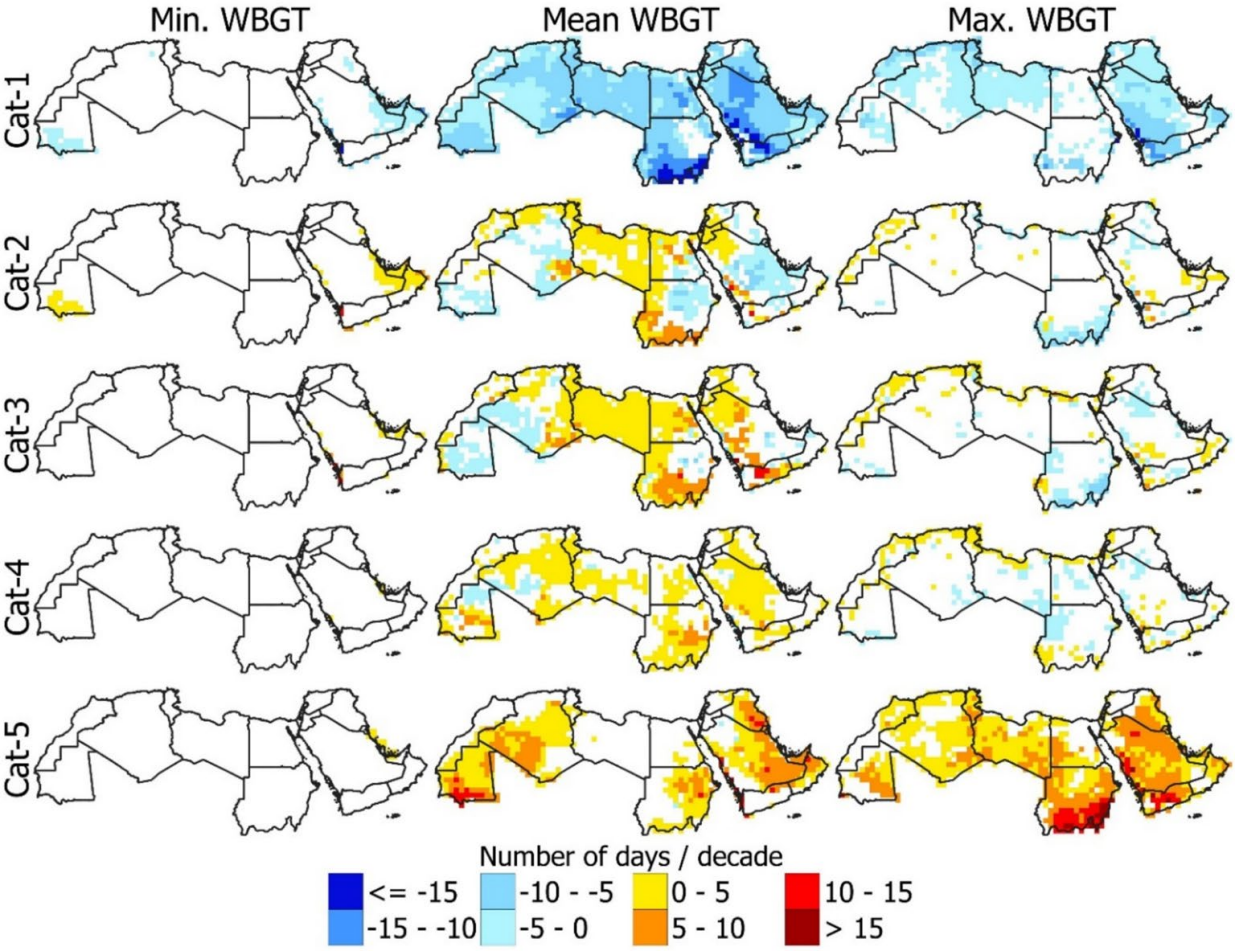
Change trends in the number of days per decade in the MENA region.

Figure 9 depicts trends in the number of days under different heat-stress conditions, based on minimum, mean, and maximum WBGT, for the entire period 1951–2020. The changes are presented per decade. The white colour indicates insignificant changes. The results reveal that changes in the minimum WBGT were not significant across all categories. There was a decrease in mean WBGT by 5–10 days/decade for Category 1 throughout the MENA region, while the other categories showed an increasing trend by 0–10 days/decade. Regarding maximum WBGT, Category 1 showed a downward trend in most MENA nations, Categories 2–4 showed insignificant trends, and Category 5 showed an upward trend.

**Fig. 10:**
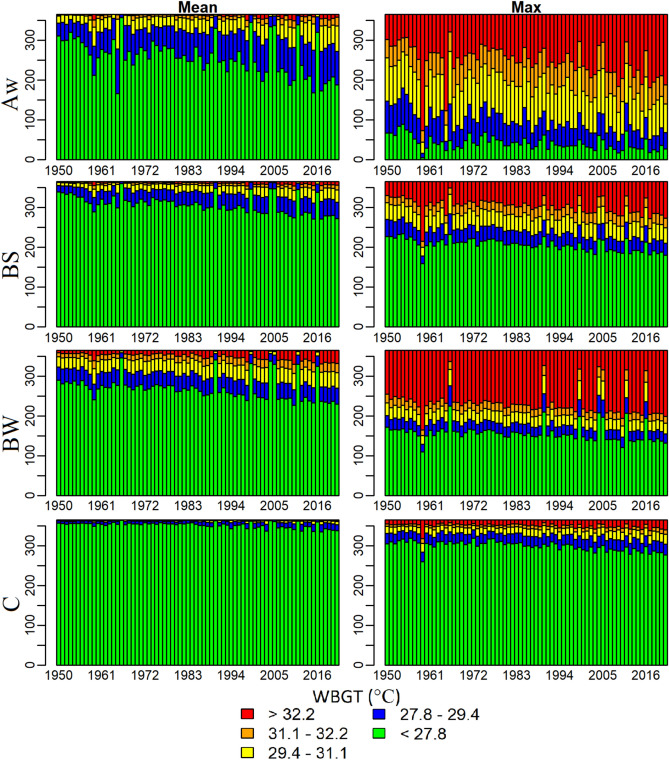
Number of annual days in different WBGT categories.

Figure 10 shows the annual number of days across different WBGT categories, based on mean and maximum values, in the climate zones of MENA (presented in Fig. 1). The columns represent climate zones, the rows represent the WBGT types, the x-axis represents the years, and the y-axis represents the annual number of days. The colours indicate various WBGT categories. The figures show a downward tssprend in normal conditions, based on mean WBGT, across most climate zones throughout the study period. In contrast, the number of moderate-to-extreme heat-stress days increased. The largest increase in extreme heat stress days was observed in the Aw and Bw zones. In contrast, the smallest decrease in normal conditions was noticed in Zone C. The changes over days under different heat stress conditions, based on the maximum WBGT, were greater than those based on the mean WBGT. However, there was a similar trend to the mean WBGT. The largest increase in days with extreme heat stress conditions was observed in Zone AW, while the smallest was in Zone C.

**Fig. 11:**
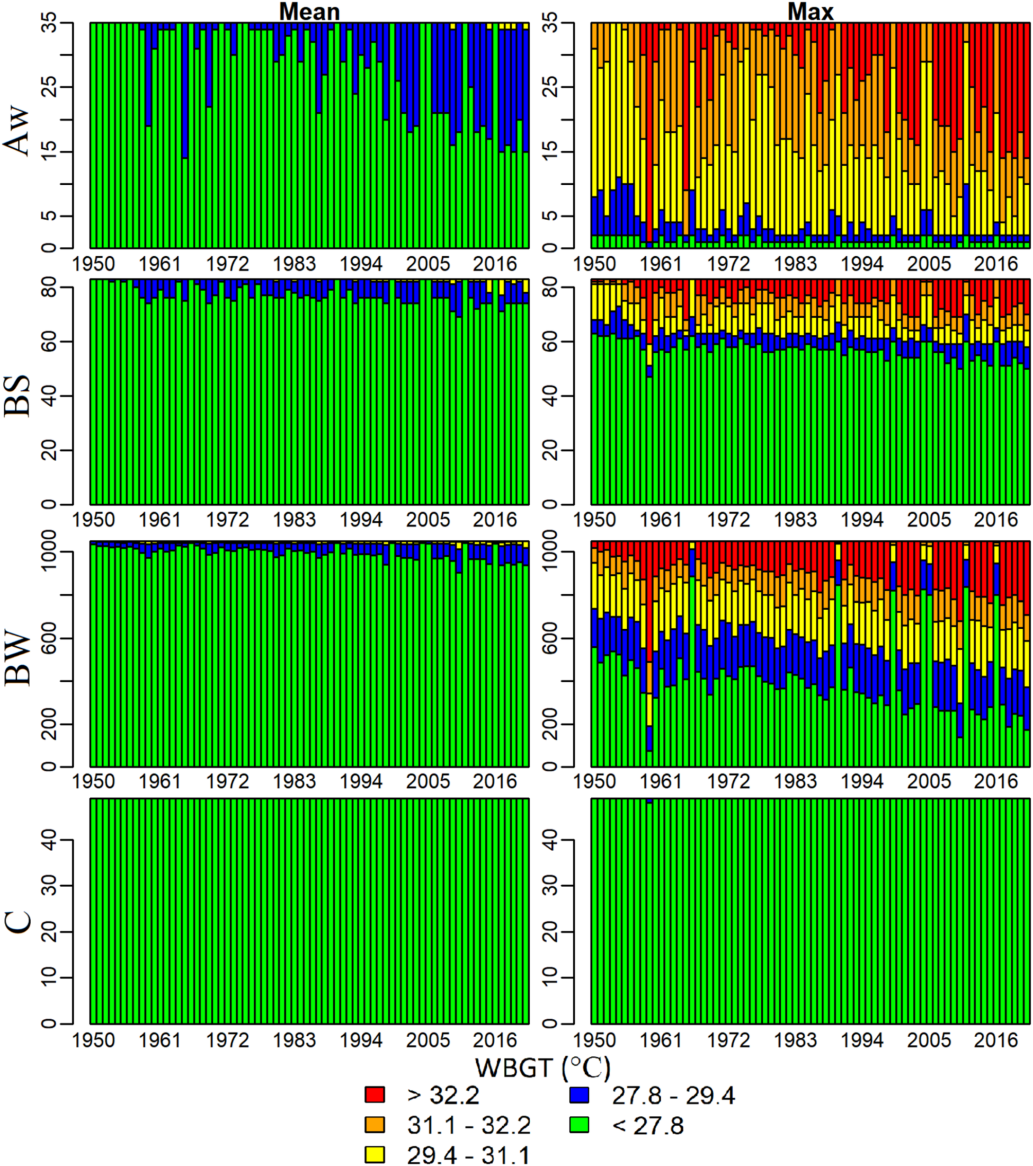
Affected areas with different categories of WBGT and different climate zones.

Figure 11 provides a quantitative assessment of the areas affected by different levels of heat stress across the four Köppen climate zones (Aw, BS, BW, and C) from 1950 to 2020 (x-axis), while the y-axis quantifies the absolute spatial extent (measured in grid points) for each category. Zone C exhibited the highest thermal stability, maintaining Cat-1 in mean and maximum WBGT conditions across nearly 100% of its spatial domain throughout the whole period. In Zone BW, the amount of land experiencing Cat-1 (Normal conditions) in maximum WBGT shrank dramatically, dropping from about 50% of the total area in the 1950s to just 20% in the most recent decade (2011–2020). Concurrently, the geographic area subjected to Cat-5 (Extreme conditions) in maximum WBGT quadrupled, expanding from 5 to 20%. The most drastic change happened in the Zone Aw. While mean WBGT conditions in the Aw zone showed a gradual contraction of the Cat-1, the maximum WBGT profile inverted completely. During the 1^st^ decade, only 14% of the Aw geographical area was classified under Cat-5 maximum WBGT conditions. By the last decade, it had expanded to dominate 43% of the zone. The data confirm that the region’s heat crisis is not just about places getting hotter. Instead, extreme heat is physically spreading to cover massive new areas across the southern and central MENA region.

**Fig. 12:**
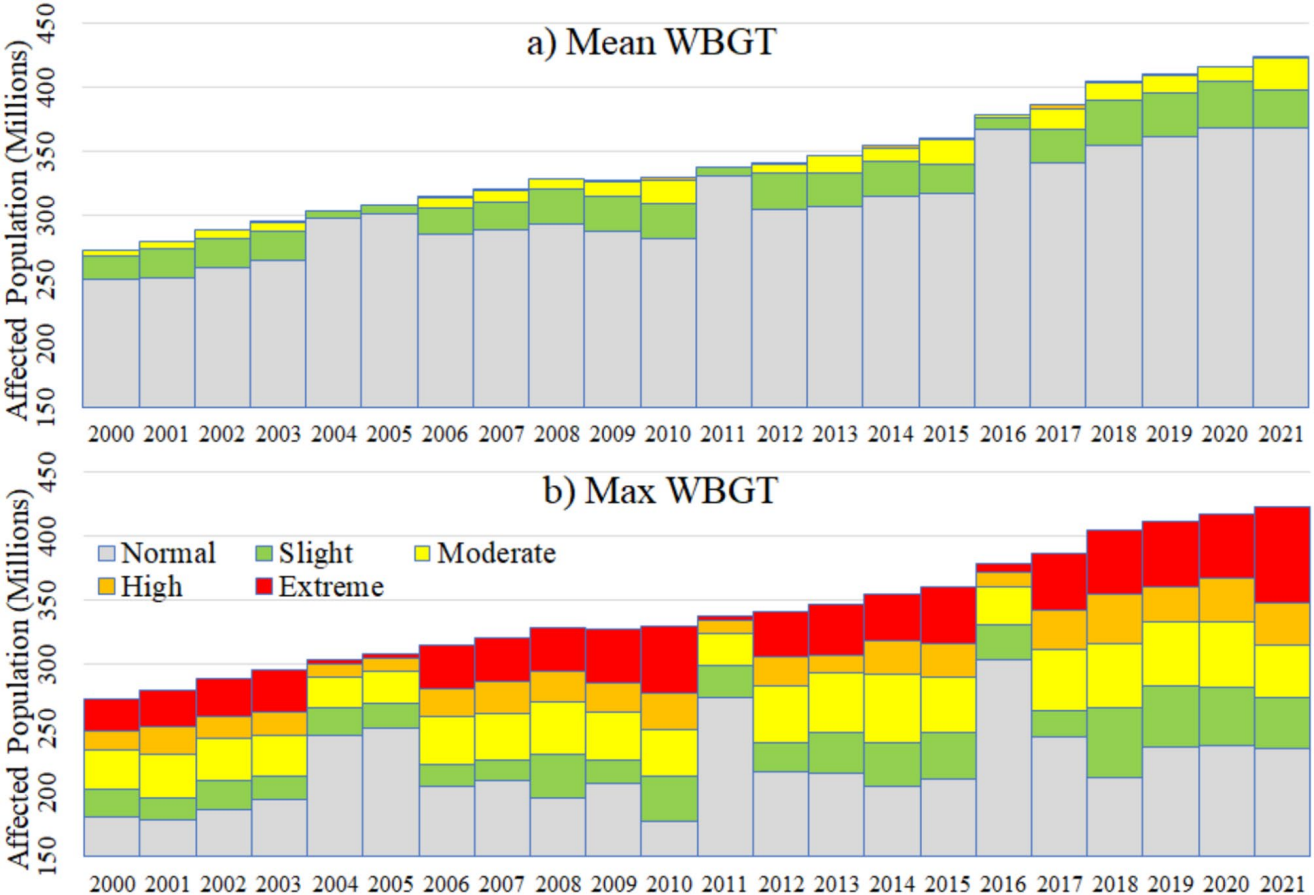
Affected population for different categories of (**a**) mean and (**b**) maximum WBGT.

Figure 12 illustrates the affected population based on the mean and maximum WBGT from 2000 to 2021. The period was limited to 2000–2021, as gridded yearly population data is available only for this periodsp. The colours represent the WBGT category. The figure shows a population increase over time in MENA. Most populations experienced normal mean and maximum WBGT conditions. In contrast, high and extreme mean WBGT conditions expose the fewest people. However, the trend for the population exposed to heat stress conditions is increasing. Table 2 shows the significant changes in annual population increment across categories of mean and maximum WBGT. It shows a yearly increase of nearly half a million people exposed to moderate heat stress based on mean WBGT. The increases were much faster for maximum WBGT. There was an annual increase of approximately 1.23 million people experiencing extreme heat stress for at least one day.

**Table 2:** Annual significant changes in population for each category of mean and maximum WBGT.

Category	Mean (Capita)	Maximum (Capita)
1	5,614,315	2,448,851
2	655,246	1,137,990
3	479,855	1,016,579
4	25,862	726,833
5	–	1,227,404

**Fig. 13:**
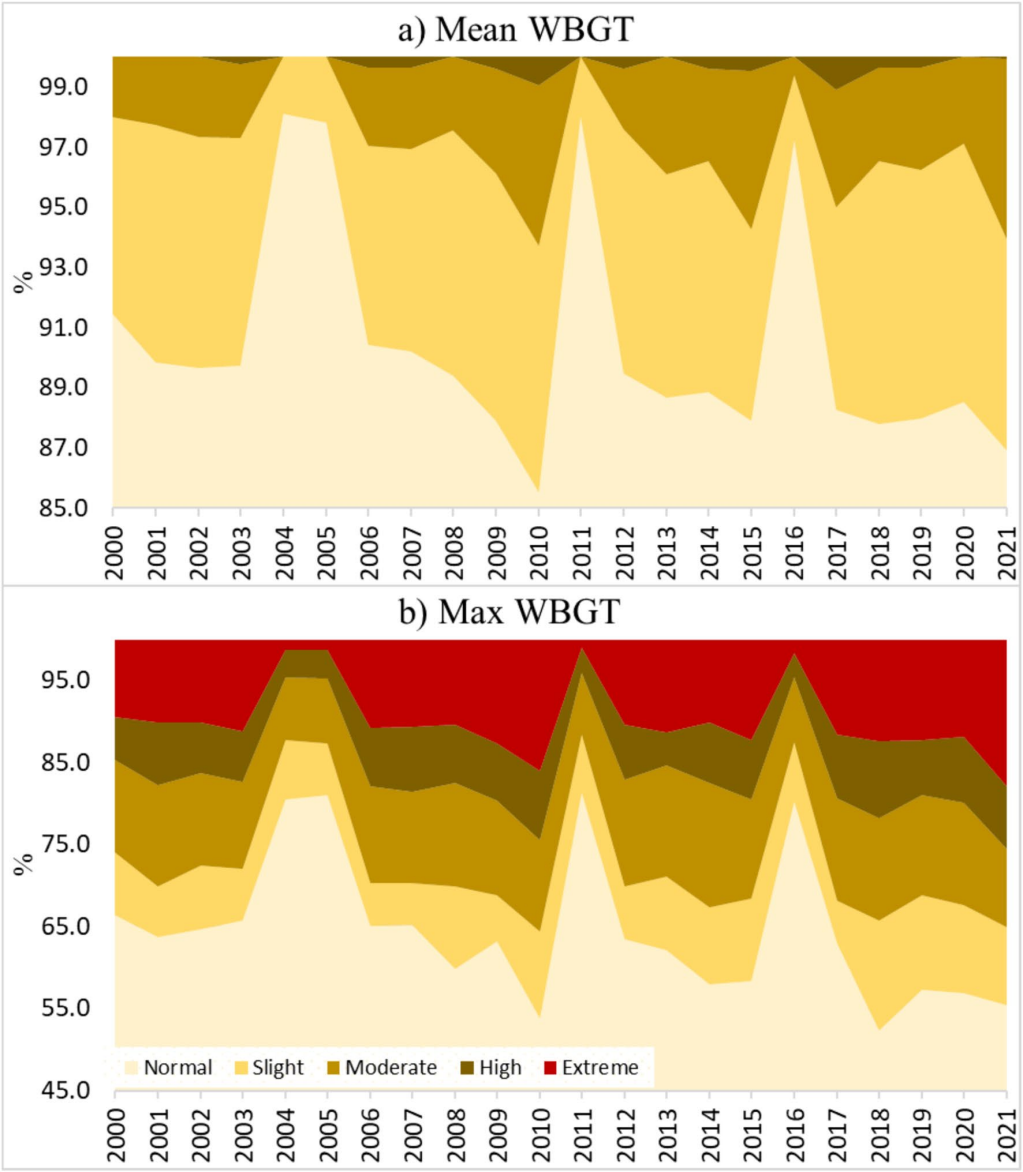
Proportional distribution of the MENA affected population to varying categories of (**a**) mean and (**b**) maximum WBGT from 2000 to 2021.

Figure 13 shows the percentage of the population affected based on the mean and maximum WBGT from 2000 to 2021, where each colour represents a WBGT category. For the mean WBGT (Fig. 13a), the vast majority of the population remains within the “Normal” category, consistently exceeding 85%. However, a gradual contraction of this baseline is evident over the two-decade period, corresponding with a proportional expansion in the “Slight” and “Moderate” exposure categories. The slight increase in the percentage of the affected population exposed to higher heat stress starts in 2010 and, with a small trend, continues until 2020. Exposure of the high-category population is minimal under the mean condition. The climatic shift is markedly more obvious regarding maximum WBGT (Fig. 13b). The proportion of the population experiencing “Normal” maximum heat conditions contracted from approximately 66% in 2000 to 55% by 2021. Concurrently, the percentage of the population exposed to “Extreme” heat stress nearly doubled over the study period, expanding from roughly 9% to approximately 18%. This proportional increase confirms that the escalating heat exposure in the MENA region is heavily driven by the intensifying severity and spatial footprint of extreme climatic conditions, rather than being solely an artefact of population expansion.

## Discussion

This study analysed several vital characteristics of WBGT in MENA: spatiotemporal trends in WBGT, the geographic area affected, and the percentage of the population experiencing varying degrees of heat stress. Findings showed that the highest WBGT was recorded in countries in the Gulf, such as the United Arab Emirates, Saudi Arabia, Kuwait, and Qatar, making them especially vulnerable to heat. This is in agreement with the published literature^57,58^. Al-Bouwarthan et al.^57^ estimated the highest hourly mean WBGT values of 31–33 °C in Saudi Arabia. Indeed, the same countries in this Gulf region have been identified by earlier studies as vulnerable to severe heat waves, which can significantly affect many socioeconomic variables, including human health and electricity consumption^58,59^. Studies have indicated that MENA has an arid and semi-arid climate, and thus it is more prone to heat stress due to high temperatures, low humidity, and high solar radiation^58^. Concerns have been increasing over the past few years about the implications of climate change on regional weather patterns and the associated heat risks^60,61^.

It further quantified the geographical spread of affected areas in MENA, specifically those where heat-stress conditions have intensified or become more frequent. Most regions in MENA showed increases in minimum, maximum, and mean WBGT values. The highest increment of 0.4 °C per decade was recorded in the eastern part, while an increment of 0.2–0.3 °C per decade was noticed in the western parts of MENA. Deviation of the daily minimum, maximum, and mean WBGT values increased by 2.3, 1.2, and 1.5 °C, respectively, over the recent decade in the eastern region, including the UAE, Saudi Arabia, Qatar, and Oman. The trend deviation was strongest in the recent decade of 2011–2020. This supports what was previously published by Almazroui^62^ and Safieddine et al.^60^. According to Almazroui^62^, the maximum and minimum temperatures increased by up to 0.71 and 0.48 °C per decade, respectively. These rising air temperatures contributed to increased WBGT, particularly in the absence of wind^63^. Also, Safieddine et al.^60^ reported a 4 °C increase in WBGT in the Persian Gulf.

The minimum WBGT does not show a clear trend across the middle region of MENA, including Egypt and Sudan. In contrast, all zones showed tendencies toward mean and maximum WBGT values of 0.2–0.3 °C. These findings agree with the results by Mostafa et al.^64^ on the increased occurrence of hot days and nights within specific zones of MENA, Nashwan et al.^65^ in Egypt, and the increased RH as reported by Kjellstrom et al.^66^.

The steeper WBGT trends observed in the eastern MENA region compared to the western sectors likely reflect differences in regional circulation patterns, moisture availability, and land–atmosphere feedback processes. The eastern Arabian Peninsula and Persian Gulf region are strongly influenced by the Shamal winds, a northwesterly low-level flow that modulates temperature, dust loading, and boundary-layer dynamics^67,68^. Variability in Shamal intensity can alter near-surface wind speeds and radiative forcing through dust–radiation interactions, thereby influencing daytime WBGT extremes. In addition, proximity to the Persian Gulf enhances low-level moisture advection, particularly during late summer, increasing humidity-driven heat stress in coastal and eastern inland regions^39^. Moisture influx from the Gulf has been shown to amplify wet-bulb temperatures and exacerbate heat stress, even when dry-bulb temperature trends are comparable to inland areas^69,70^. Large-scale climate variability may also contribute to spatial heterogeneity in WBGT trends. Teleconnection patterns such as ENSO influence temperature and precipitation variability across parts of the eastern Mediterranean and the Arabian Peninsula by modulating regional circulation anomalies^71,72^. Such variability can affect humidity transport, cloud cover, and wind regimes, thereby indirectly shaping the evolution of long-term heat stress.

The changes in the number of days, by each WBGT category defined by the US Department of the Army, in most cases indicate an increase compared with the reference period 1951–1980. In particular, in the southeastern part of the MENA region, there has been a registered increase in the number of days in categories 3, 4, and 5 for both maximum and mean WBGT. Sudan and Yemen, like many regions worldwide, have experienced exposure of their populations to moderate-to-extreme WBGT conditions, which increases the risk of heat-related diseases. The study showed the largest increase in the exposed population to extreme heat stress in Zone Aw, along the Persian Gulf. This is due to a high increase in temperature-related extremes in this populated region. Tanarhte et al.^73^ reported the largest increases in daytime temperatures and heat wave frequency in the Persian Gulf region. Pal and Eltahir^39^ projected an exceedance of WBGT, a critical threshold for human tolerance in the Persian (Arabian) Gulf, using Regional Climate Models.

Climate change is exacerbating heat exposure in the MENA region and across the world. The Arabian Gulf, specifically, is projected to experience intolerable temperatures in the near future^39^. Recent studies have shown that if global warming continues unabated, it will have a toll on the health and labour productivity of the citizens in the MENA region. For instance, a rise of 1 °C in global temperatures can reduce labour productivity by 0.57%^74^. Global warming is likely to exacerbate rising temperatures in the MENA region, thereby worsening health conditions among its citizens and increasing the burden of disease. Such an increase in health risks can have significant impacts on the national economy of the countries in the MENA region^75^. Using the most updated climatology data, the development of such high-resolution WBGT maps can help formulate region-specific development and heat plans that take into consideration the purposes of limiting adverse repercussions of rising temperatures and adjusting strategies aimed at mitigating climate change to secure the safety of life and work for the vast population that inhabits the MENA region. Moreover, they may help in developing a climate-resilient economy and infrastructure of the region.

In this study, ERA5 data were used to assess how WBGT varied over time. A few studies^76,77^ identified anomalies and errors related to the ERA5 dataset. Some key climatic variables, such as temperature, humidity, wind speed, and precipitation, have been reported inconsistently, as noted in a few studies, including^77,78^. These errors are likely to affect the calculations and interpretations in thermal stress research. LandScan’s gridded population data also carries similar uncertainty. It usually gives misleading estimates of the local population in densely populated areas^79^. Such research outcomes must therefore be interpreted with caution. The results should therefore be carefully considered and analysed by both the researchers and the readers.

While ERA5 and LandScan are robust global datasets, their application in hot-arid regions introduces specific quantitative uncertainties. Recent research by Kong & Huber^51^, using the ERA5 reanalysis, shows that in subtropical dry regions (the Middle East), heat stress metrics are highly sensitive to the covariation between wind speed and solar radiation. Specifically, for extreme heat stress, reanalysis-based calculations can underestimate WBGT by 6 to 10˚C in arid environments due to strong solar radiation and low-wind conditions. These errors in ERA5’s representation of extremes suggest that our findings may represent a conservative estimate of peak thermal stress. LandScan introduces spatial uncertainty by modelling ambient populations rather than strict residential counts. While highly precise, validation in arid zones suggests local variances of roughly 16% in populated sub-provinces, with potential overestimation of exposure in well-lit desert infrastructure^80^.

WBGT is a composite thermal index that integrates air temperature, humidity, wind speed, and radiation using physically based energy-balance equationss^43^. Changes in WBGT therefore reflect combined variations in dry-bulb temperature, vapour pressure (or dew point), wind-driven convective heat exchange, and shortwave and longwave radiation fluxes. Although this study identifies statistically significant changes in WBGT categories and the affected population, a full quantitative attribution of the relative contributions of different variables was beyond the scope of the present work. Previous studies suggest that long-term increases in air temperature generally dominate mean WBGT trends, while radiation and wind variability disproportionately influence extreme WBGT values^25,81–83^. Studies also showed that in subtropical dry regions, daytime heat stress is highly sensitive to radiation–wind interactions, whereas humidity exerts stronger control incoastal and humid climates^84,85^, which may explain the stronger maximum WBGT trends in eastern MENA than the minimum trends. An enhanced mechanistic understanding is possible by applying formal variance decomposition or sensitivity analysis techniques, which would allow isolation of the proportional contribution of each meteorological driver to observed WBGT changes across MENA. Future research should therefore implement component-wise attribution frameworks, such as perturbation-based sensitivity analyses or regression-based decomposition approaches, to quantify the extent to which the observed increase in WBGT is attributable to thermodynamic warming driven by changes in humidity, radiation fluxes, or wind patterns.

## Conclusion

In this respect, this study fully assessed temporal and spatial changes in outdoor WBGT, the extent of the area and population exposed to different levels of heat stress across the Middle East and North Africa (MENA). WBGT was projected in this study using the widely adopted Liljegren method, based on the latest meteorological data from the ERA5 dataset. Heat exposure was also computed using LandScan population data. The results show a sharp increase in heat stress across the MENA region. The rise in temperature increased the number of hot days. Throughout much of the MENA region, the number of "normal heat days" each year is plummeting, while the number of "severe and extreme heat stress days" is soaring. The study also found that the areas most affected by extreme heat waves are expanding. This rise reflects more people being exposed to more significant heat stress. The results show that heat stress risk is increasing in the MENA region and underscore the need for effective countermeasures. These results contribute to the scientific literature on climate change and related impacts, particularly regarding heat surge events. A geographic area and population exposure assessment might give insights into the area affected by the disaster and the population exposed to different levels of heat stress, enabling the identification of hotspots and susceptible populations in need of urgent care. This information could contribute to monitoring, anticipating, and managing thermal stress conditions in the MENA region by setting research objectives, designing multi-disciplinary studies, and formulating novel methodologies. In this regard, ERA5 data were used in this study, which showed inconsistencies and inaccuracies in WBGT assessments in certain regions. The available observation data can be used in the future to verify the findings. This can also be used to bias correct ERA5 data to better estimate WBGT changes in MENA.

## Figures and Tables

**Fig. 14:**
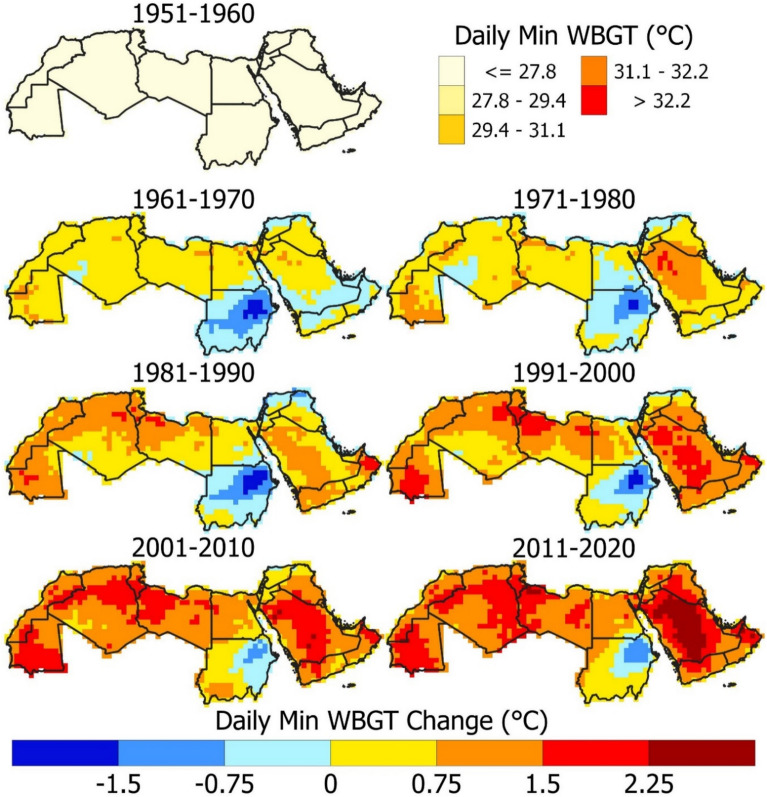
Spatial distribution in the historical daily minimum WBGT in the MENA region under various thermal conditions.

**Fig. 15:**
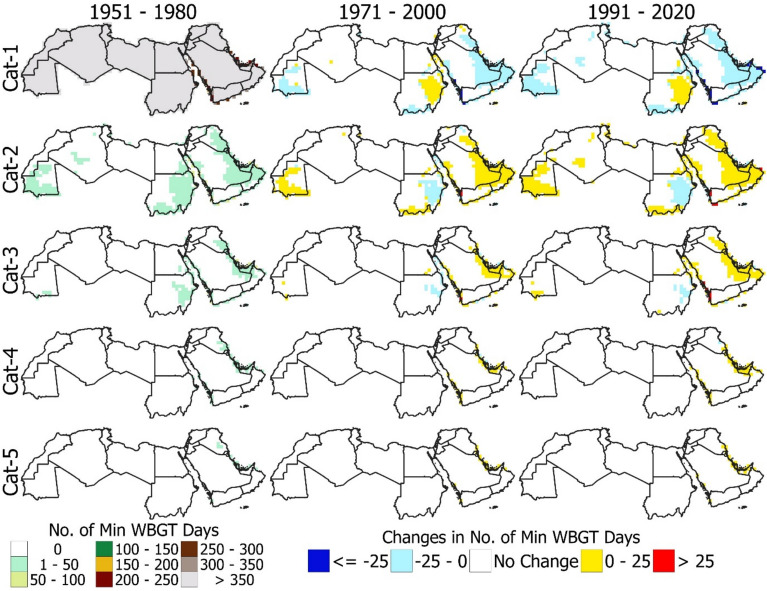
The annual number of minimum WBGT days for each category in the MENA region.

## Data Availability

The authors declare that the underlying data used to support the findings of this study are available from the corresponding author upon reasonable request.

## Appendix

See Figs. 14 and.

**Table 3:** Heat Categories definition based on WBGT classification^86,87^.

Definition	Heat category	WBGT class (°C)
Normal conditions	1	25.6 to 27.8
Less than the ideal conditions	2	27.8 to 29.4
Moderate risk of heat-related illness	3	29.5 to 31.0
High risk of heat-related illness	4	31.1 to 32.2
Extreme conditions	5	> 32.2

## Code availability

The authors declare that the underlying data used to support the findings of this study are available from the corresponding author upon reasonable request.
